# National

**Published:** 1897-08

**Authors:** 


*National.* Secretary Wilson has taken up the subject of scab in sheep, which has been so thoroughly agitated by some of the State veterinarians in the West, not because this disease is more widespread than before, but for the reason that the centres of its existence are well-known, the methods of its extension better understood, and what is necessary for its control well defined; and, acting on this information, he has given public notice to all common-carriers that it is a violation of the law to receive or transport them. When discovered, thorough disinfection of all cars, pens, etc., must immediately follow.

